# Stability and Induced Magnetism by Edge Modification of HfS_2_ Nanoribbons

**DOI:** 10.1021/acs.langmuir.5c02830

**Published:** 2025-07-29

**Authors:** Bruno G. A. Pimenta, Railson da Conceição Vasconcelos, Pedro H. de Oliveira Neto, Rafael F de Menezes, Kayla G. Sprenger, Ricardo Gargano

**Affiliations:** † Institute of Physics, 28127 University of Brasília, Brasilia, DF 70910-900, Brazil; ‡ Department of Chemical and Biological Engineering, 122701 University of Colorado Boulder, Boulder, Colorado 80309-0401, United States

## Abstract

The development of two-dimensional (2D) structures has had an immense impact on the field of nanoelectronics. However, many potential candidates for practical applications remain unexplored. One such underinvestigated group is HfS_2_ nanoribbons. In this study, we aimed to assess the influence of nanoribbon geometries (armchair or zigzag) on key properties such as stability and band gap. Additionally, we explored the potential for edge-modification-induced magnetism. These investigations were conducted using first-principles calculations based on density functional theory (DFT). Our findings demonstrate that all simulated systems are thermodynamically stable and some also exhibit dynamical stability. In terms of band structure, the armchair configuration behaves as a semiconductor, while the zigzag configuration varies between semiconducting, metallic, and half-metallic depending on the edge characteristics. Apart from minor variations in band gap values, the ribbon’s general properties remain consistent as their width changes. Most notably, we observed induced magnetism in HfS_2_ nanoribbons through edge modifications, which transformed nonmagnetic ribbons into magnetic ones. Consequently, we demonstrate that HfS_2_ nanoribbons are promising candidates for applications in both nanoelectronics and spintronics.

## Graphical abstract

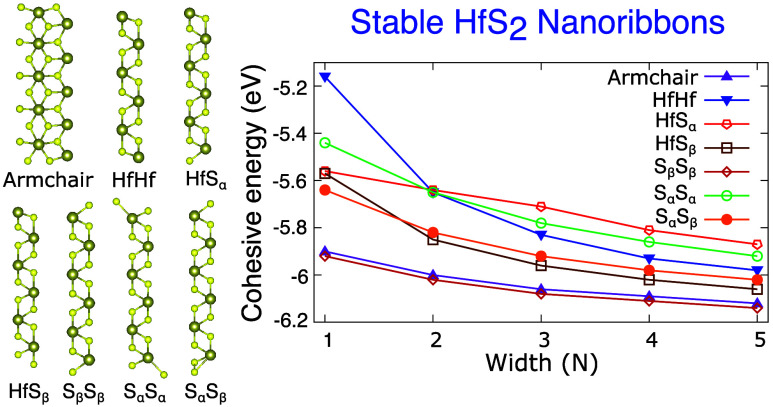

## Introduction

The successful isolation of graphene monolayers^1^ challenged classical physics principles from the early 20th century,^2^ defying the widely accepted belief that two-dimensional materials could not exist in a stable, free-standing form. Since then, 2D materials have been extensively studied. These materials are known for their remarkable electromagnetic properties,^3^ enabling significant technological innovations.^4,5^ To date, devices such as sensors and transistors have been revolutionized using such low-dimensional materials, becoming more sensitive, compact, and efficient.^6,7^

A prominent subset of two-dimensional materials is transition-metal dichalcogenides (TMDs). These materials typically exhibit desirable properties, including moderate band gap values, chemical stability, and high mobility at room temperature.^8–11^ They also have potential applications in catalysis, photovoltaic cells, and high-performance transistors.^12–15^ One example is the HfS_2_ monolayer, and recent advances in experimental techniques have led to various methods, such as mechanical exfoliation and chemical vapor deposition (CVD), for synthesizing ultrathin HfS_2_ films while preserving their crystal quality.^16–18^

Materials intended for spintronics applications ideally exhibit both half-metallic states, which allow for spin-polarized current, and ferromagnetism, which maintains alignment. In its pristine form, the HfS_2_ monolayer is a nonmagnetic semiconductor,^19^ lacking both ferromagnetism and half-metallic characteristics, limiting its direct suitability for spintronics. However, several studies have demonstrated that intrinsic ferromagnetic states can emerge in TMDs like HfS_2_ through methods such as transition-metal doping and defect engineering.^20,21^ Specifically, dangling bonds can arise from specific edge configurations of graphene fragments, which induce a nonzero magnetic moment.^22,23^ In this context, modifying the edges of the two fundamental nanoribbon configurations derived from the HfS_2_ monolayer, namely, “armchair” and “zigzag,” represents an underexplored strategy that could also potentially achieve these key spintronic properties.

Motivated by these considerations, we evaluated the properties of pristine armchair and zigzag HfS_2_ nanoribbons using *ab initio* calculations. Specifically, we investigated their structural stability and band structure configurations. Additionally, we examined the effects of edge modifications and variations in ribbon width across all possible edge terminations. Most notably, we found that certain edge configurations can indeed induce magnetic states, providing a path toward enhancing the suitability of this promising material for spintronics.

## Computational Details

Geometric optimization and total energy calculations were performed using spin-polarized density functional theory (DFT) as implemented in the SIESTA package.^24^ The exchange-correlation functional was treated using the Perdew–Burke–Ernzerhof (PBE) method within the generalized gradient approximation (GGA).^25^ We employed Troullier–Martins pseudopotentials,^26^ a double-ζ polarized (DZP) basis set, a mesh cutoff of 380 Ry for the real-space grid, and a convergence threshold of 10^–5^ eV for the total energy. The nanoribbons were modeled with periodic boundary conditions along the *z*-axis with a vacuum spacing of more than 20 Å in the *x* and *y* directions to eliminate self-interactions between mirror-image ribbons. All structures were fully relaxed, until the forces on each atom were less than 0.005 eV/Å. The necessary integrals for the total energy and the partial density of states (PDOS) were calculated using a 1 × 1 × 10 and a 1 × 1 × 80 *k*-point grid, respectively, both generated with the Monkhorst–Pack scheme.^27^ We further conducted convergence tests using various combinations of pseudoatomic orbitals and real-space grid cutoffs to ensure the robustness of our calculations.

A first evaluation of stability is made with the computation of the cohesive energies.^28^ To further establish the overall stability of the simulated structures, we calculated the phonon band structure using a 0 × 0 × 3 supercell in SIESTA’s Force Constant (FC) computation. The lattice vibration frequencies were subsequently determined using SIESTA’s *vibra* utility.

Additionally, to gain a deeper understanding of the discretization and confinement effects in the extracted ribbons and to validate our methodology, we simulated the monolayer form of HfS_2_, comparing its band structure and phonon dispersion to previously published results.^16,29,30^ In this case, we used a 10 × 1 × 10 grid for the total energy calculation and an 80 × 1 × 80 grid for the PDOS calculation. Meanwhile, a 3 × 0 × 3 supercell was used for FC computation. Our results showed close agreement with previous findings.

## Results and Discussion

Since HfS_2_ monolayers have been previously studied and even experimentally obtained,^16–18^ we first evaluate some general electronic and structural parameters obtained using our aforementioned approach. In Figure 1a, we present the optimized monolayer. The lattice constants determined in this work are *a* = *b* = 3.64 Å, consistent with values reported in the literature.^16^ Furthermore, Figure 1b illustrates the vacuum region of *c* = 20 Å, used throughout this work to isolate lower-dimensional structures.

**Figure 1:**
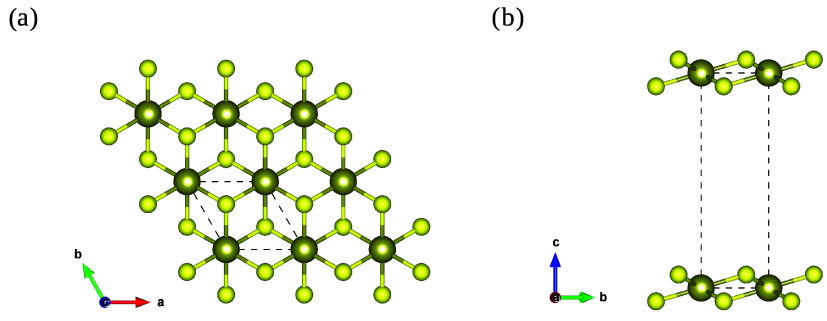
Atomic structure of the HfS_2_ monolayer with lattice vectors. (a) Top and (b) side views showing the vacuum spacing between adjacent layers.

Further characterization of the monolayer is performed, with the results shown in Figure 2. Specifically, inspection of the electronic band structure along the reciprocal path Γ–*M*–*K*–Γ and PDOS in Figure 2a,2b confirms the semiconductor behavior of this material. Moreover, the band gap is found to be 1.28 eV, which is slightly smaller than the values reported in the literature.^16,29,30^ This discrepancy arises from the choice of functional, which is known^31–35^ to underestimate this quantity. Regarding its overall stability, the phonon band structure in Figure 2c reveals no negative frequencies (no imaginary frequency modes), suggesting that the material can be considered dynamically stable. Furthermore, its overall shape matches the results found in the literature.^30^

**Figure 2:**
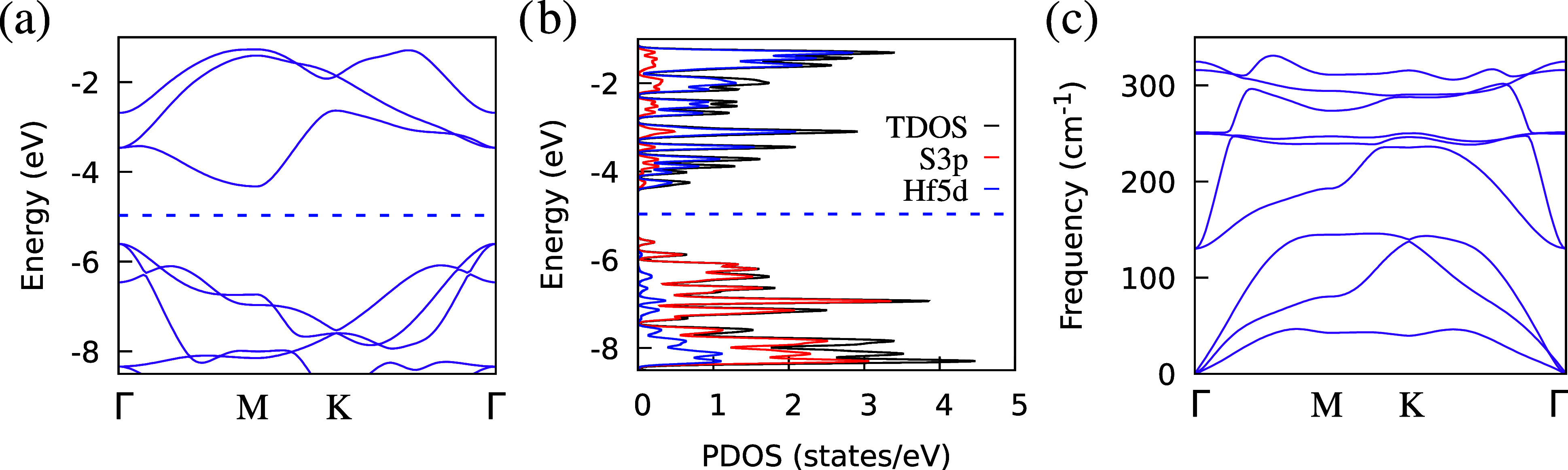
Electronic and structural results for the HfS_2_ monolayer. (a) Electronic band structure, (b) projected density of states (PDOS), and (c) phonon band structure. The reciprocal path Γ–*M*–*K*–Γ is used in (a, b).

To determine the properties of the nanoribbons, it is crucial to first define all possible edge configurations that can be derived from the stable HfS_2_ monolayer. TMD-based monolayers are commonly known to belong to the *P*3̅*m*1 space group.^4,11,36^ Therefore, when the nanoribbons are extracted, the original symmetry is broken in specific ways, resulting in two main types of edges: an armchair and a zigzag.

The symmetry breaking in armchair-type nanoribbons allows for only one possible edge configuration. Therefore, in this arrangement, there is only a single armchair-HfS_2_ nanoribbon (AHfS_2_), as illustrated in Figure 3a. In contrast, zigzag-HfS_2_ nanoribbons (ZHfS_2_) can be categorized based on the arrangement of sulfur (S) atoms at the ribbon’s edges. We introduce the following notation to describe these zigzag configurations based on the number of S atoms at the edge of the supercell: S_α_ for two sulfur atoms and S_β_ for one sulfur atom. Additionally, a zigzag configuration without sulfur atoms at the edge is represented solely by hafnium (Hf). By combining these three types of edges, we identified a total of six zigzag forms. These are shown in Figure 3b through 3g, and are named, respectively, HfHf, HfS_β_, HfS_α_, S_α_S_α_, S_α_S_β_, and S_β_S_β_.

**Figure 3:**
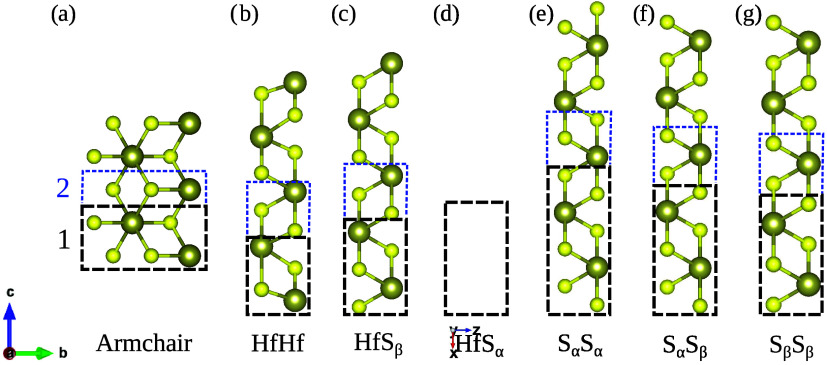
Graphical illustration with reference basis of the supercells of unoptimized nanoribbons extracted from HfS_2_ in monolayer form. Following our notation, the respective ribbons are obtained: (a) armchair, (b) HfHf, (c) HfS_β_, (d) HfS_α_, (e) S_α_S_α_, (f) S_α_S_β_, and (g) S_β_S_β_. Forms b–g constitute the set of zigzag ribbons. The black dashed rectangles represent the supercells of the narrowest ribbons (*N* = 1), while the blue ones indicate a unit increase in width (*N* = 2). The periodicity of the structures is along the direction defined by the lattice vector **b**, while the ribbons depicted have a fixed width of *N* = 4. Hf and S atoms are represented by larger and smaller spheres, respectively.

After establishing the edge arrangements, we conventionally define the ribbon width by counting the successive transversal insertions of HfS_2_ atoms to the narrowest supercell (*N* = 1) maintaining the edge type.^37,38^ Such a convention is illustrated by the dashed rectangles in Figure 3 where periodicity is along the lattice vector **b** and vacuum is inserted along the vectors **c** and **a**. Within this convention, the ribbons are essentially one-dimensional. The lattice constant is then determined by the optimized distance between equivalent atoms separated by one spatial period,^39,40^ which equals 6.3 Å for the armchair-type and 3.6 Å for the zigzag-type. Thus, the nanoribbons presented in Figure 3 correspond to supercells with *N* = 4. This choice enabled us to investigate the influence of the edge type and ribbon width on thermodynamic stability. The cohesive energies (*E*_*c*_) of the supercells are calculated as

1 $$E_{c}=\frac{E_{T}\left[\mathrm{Hf}S_{2}\right]-n_{\mathrm{Hf}}E_{\mathrm{Hf}}-n_{S}E_{S}}{n_{\mathrm{Hf}}+n_{S}}$$

where *E*_T_ is the total energy of the supercell, *n*_Hf_ and *n*_S_ are the numbers of each species, and *E*_Hf_ and *E*_S_ are the total energies of the isolated atoms. Using this formula, we generated the graph in Figure 4, which illustrates the influence of width and edge type on the ribbon’s stability. All ribbons appear to be thermodynamically stable after optimization, with AHfS_2_ and S_β_S_β_ being the most stable. As expected, the stability increases with width, converging toward the cohesive energy of the monolayer at −6.21 eV. With regard to dynamical stability, only the S_α_S_β_ ribbon exhibited strictly positive phonon frequencies, indicating a stable configuration. This result was expected, given the well-known instability of unpassivated nanoribbons.^41,42^

**Figure 4:**
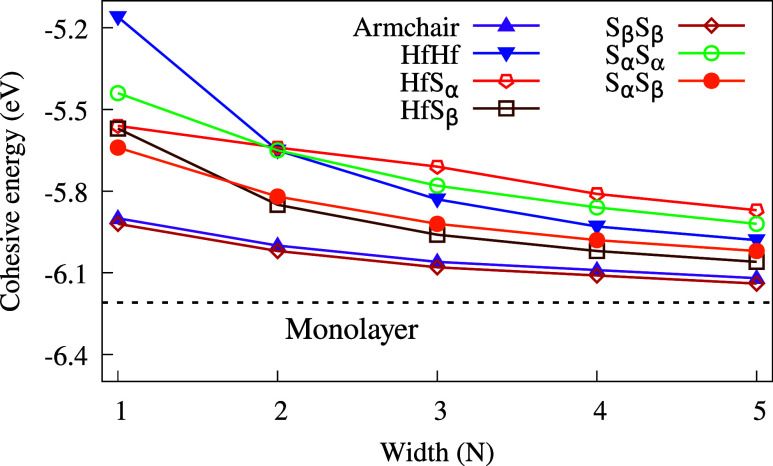
Cohesive energies per unit cell (*E*_c_) as a function of the ribbon width. The dashed line indicates the cohesive energy for HfS_2_ in the monolayer form.

Additionally, we analyzed the band structures of each ribbon with widths ranging from *N* = 1 to *N* = 5. Except for small variations in the band gap values, as shown in Figure 5, the overall characteristics of the band structures are preserved. Thus, we selected the representative width of *N* = 5 to present the band structures and the total and partial densities of states (TDOS and PDOS) for both nonmagnetic and magnetic ribbons in Figures 6 and 7, respectively.

**Figure 5:**
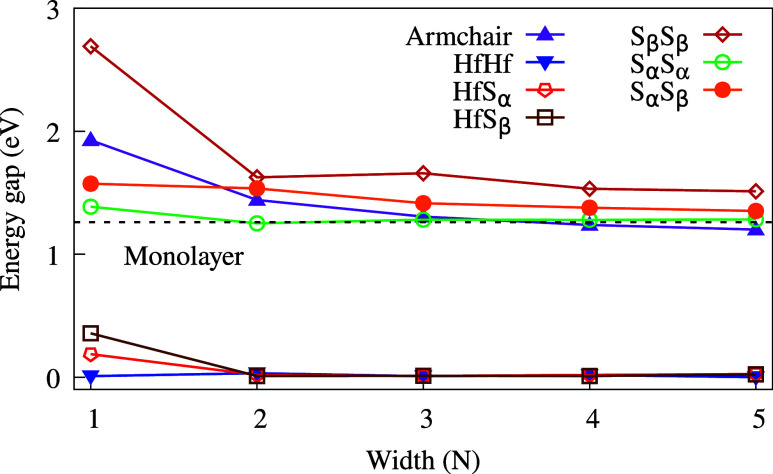
Energy gap values (*E*_gap_) as a function of the ribbon width. The dashed line indicates the energy gap for HfS_2_ in the monolayer form.

**Figure 6:**
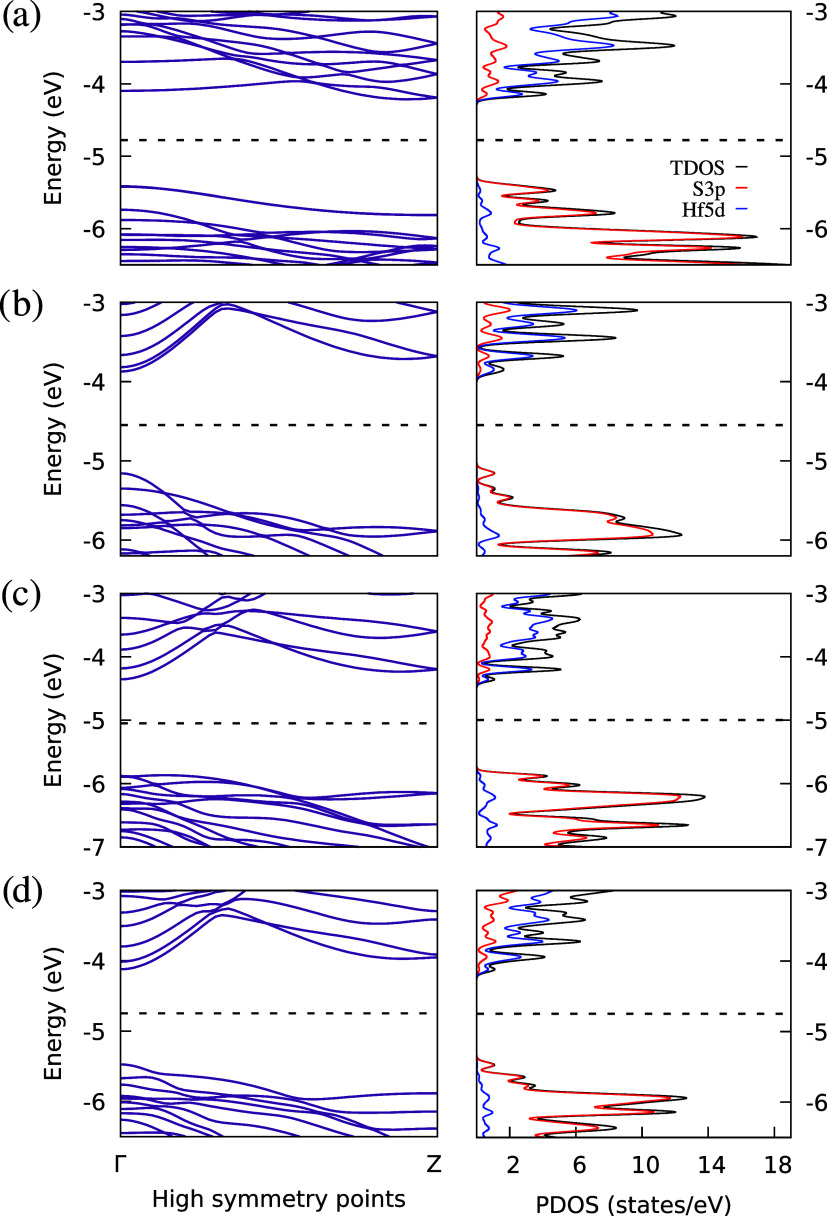
Band structure (left) and partial density of states (PDOS, right) of the nonmagnetic ribbons: (a) armchair, (b) S_α_S_α_, (c) S_β_S_β_, and (d) S_α_S_β_. Dashed lines indicate the Fermi energy. The Brillouin zone path follows Γ–*Z*.

**Figure 7:**
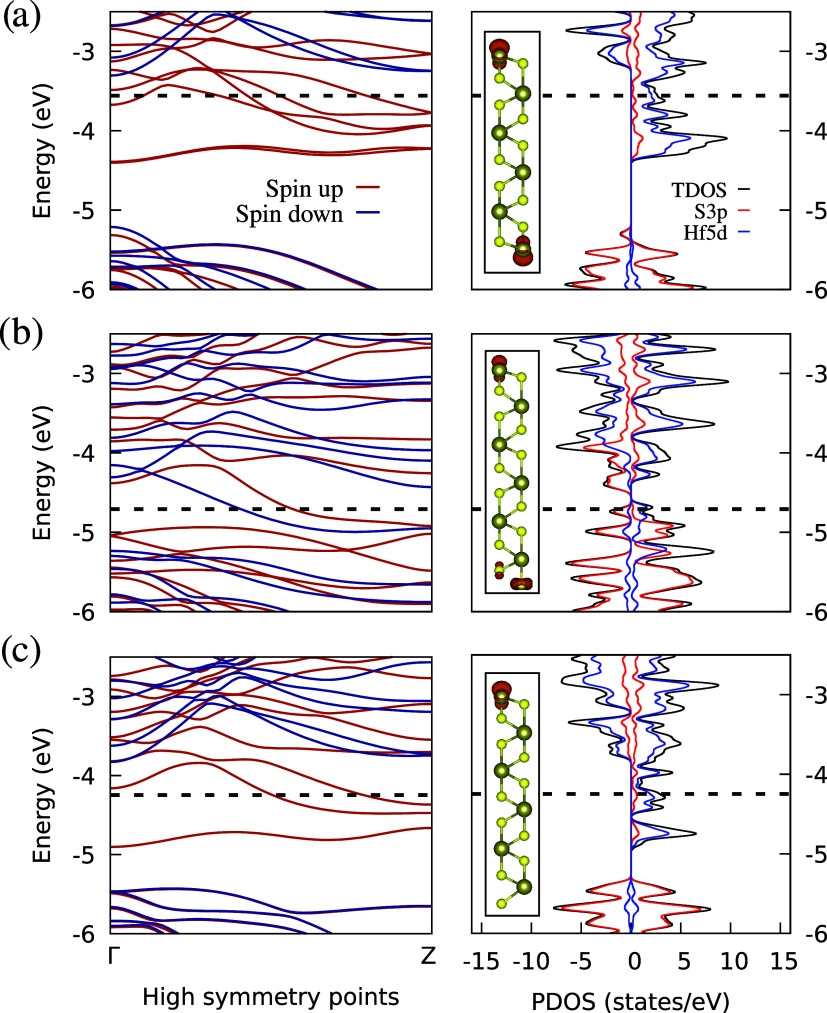
Band structure (left) and partial density of states (PDOS, right) of the magnetic ribbons: (a) HfHf, (b) HfS_α_, and (c) HfS_β_. Dashed lines indicate the Fermi energy. The Brillouin zone path follows Γ–*Z*. The insets on the right plots exhibit net spin densities, with a surface isovalue of 0.02 e·Å^–3^.

For the ribbons shown in Figure 6 (left plots), no spin polarization was observed, allowing us to classify the armchair, S_α_S_α_, S_β_S_β_, and S_α_S_β_ nanoribbons as nonmagnetic. These nanoribbons are semiconductors with band gaps as large as 1.6 eV for the S_β_S_β_ ribbon and as small as 1.2 eV for the armchair ribbon. Except for the armchair ribbon, which has an indirect band gap, all ribbons exhibit direct band gaps. In the PDOS plots (Figure 6, right), the top of the valence band is predominantly composed of sulfur 3p (S3p) orbitals, while the bottom of the conduction band primarily consists of hafnium 5d (Hf5d) orbitals. This is consistent with the valence orbitals of the individual atoms in transition-metal dichalcogenides.^38^ Although the functional used may underestimate the band gaps,^31–35^ the band structure remains qualitatively consistent with that of the monolayer when more appropriate functionals are applied.^29^ Therefore, the band structures of our ribbons are well-defined, despite potential systematic errors.

A similar analysis was conducted for the magnetic ribbons, as shown in Figure 7. As before, S3p orbitals dominate the top of the valence band, while Hf5d orbitals dominate the bottom of the conduction band (right plots). Moreover, spin polarization occurs, as observed by the asymmetrical PDOS between spin-up and spin-down channels. Hence, we classify the HfHf and HfS_β_ nanoribbons as half-metals, and the HfS_α_ nanoribbon as ferromagnetic. Our results reveal the emergence of magnetism due to spin polarization. A more precise characterization of this effect is provided by the insets in the PDOS plots of Figure 7, which show the net spin densities, represented as dark orange clouds, for each edge arrangement. It confirms the presence of dangling bonds at the edges of the ribbons,^43,44^ which produce a localized nonzero magnetic moment, analogous to magnetic graphene fragments.^22^

Table 1 summarizes the key structural, electronic, and magnetic properties of the HfS_2_ nanoribbons. HfHf and HfS_β_ stand out as promising candidates for spintronic applications, combining half-metallic states with significant magnetic moments. Notably, HfHf exhibits a substantial magnetic moment of 4 μ_*B*_, surpassing similar two-dimensional structures.^38,45–48^

**Table 1:** Electronic, Magnetic, and Stability Parameters for the Simulated Nanoribbons with Width *N* = 5^a^

			*E*_gap_ (eV)		
state	ribbons	*E*_c_ (eV)	spin up	spin down	μ (μB)
	HfHf	–5.98	0.0	1.9	4.0
magnetic	HfS_α_	–5.87	0.0	0.0	2.7
	HfS_β_	–6.06	0.0	1.6	2.0
nonmagnetic	S_α_S_α_	–5.92	1.3	1.3	0.0
	S_β_S_β_	–6.14	1.5	1.5	0.0
	S_α_S_β_	–6.02	1.4	1.4	0.0
	armchair	–6.12	1.2	1.2	0.0

a *E*_c_, *E*_gap_, and μ represent the supercell’s cohesive energy, band gap energy, and total magnetic moment, respectively.

## Conclusions

In this study, we comprehensively explored the characteristics of pristine HfS_2_ nanoribbons with all possible edge terminations. Structural optimizations and cohesive energy calculations reveal that all nanoribbon configurations exhibit considerable thermodynamic stability. However, only the S_α_S_β_ ribbon also demonstrates dynamical stability, making it the most stable structure overall.

Additionally, our results highlight the potential for tailoring HfS_2_ nanoribbons to achieve desirable properties. Specifically, starting from the pristine monolayer form, seven nanoribbons with distinct edge configurations can be derived. While the monolayer itself is a semiconductor, three nanoribbons (HfHf, HfS_β_, and HfS_α_) exhibit varied electronic properties, ranging from half-metallic to metallic. Most notably, magnetic states emerge as the edges of the ribbons are modified, demonstrating the potential for inducing magnetism through edge modification. These findings position this set of nanostructures as promising candidates for spintronic applications.
